# Research on improving the ranging accuracy of ships with stereo vision through Kalman filter optimization

**DOI:** 10.1371/journal.pone.0305714

**Published:** 2024-11-05

**Authors:** Zhongbo Peng, Jie Han, Liang Tong, Lumeng Wang, Dan Liu, Shijie Sun

**Affiliations:** School of Shipping and Naval Architecture, Chongqing Jiaotong University, Chongqing, China; Kocaeli University, TÜRKIYE

## Abstract

The real-time and accurate detection and ranging of ships play a pivotal role in ensuring navigation safety, this study aims to enhance the navigation safety and environmental perception capabilities of inland waterway vessels. In the ship detection stage, addressing challenges such as large parameters, high computational complexity, and poor real-time performance in existing ship detection models, this paper proposes the MS-YOLOv5s ship target detection algorithm. This algorithm, based on YOLOv5s, utilizes the lightweight MobileNetV3-Small network to replace the original YOLOv5s feature extraction backbone network, thereby improving the detection speed. The results indicate that the parameter size of the MS-YOLOv5s model is 3.55M, only 50.49% of YOLOv5s. Achieving a detection rate of 50.28 FPS, the precision is 96.80%, and the mAP is 98.40%, striking a balance between high accuracy and low computational demand. In the depth estimation stage, influenced by the environment, leading to unstable measurement data, this paper proposes a binocular Kalman filter fusion ranging algorithm. The standard deviation of the ranging results is minimized to 6.032μm, which is one order of magnitude smaller than traditional ranging algorithms, significantly enhancing the robustness of the measurement results. Within a distance of 20m from the ship target, the error can be controlled within 3%, showcasing the applicability of the method proposed in this paper in complex inland waterway environments contributes to the enhancement of ships’ environmental perception capabilities and navigation safety, holding positive implications for the development of intelligent vessels.

## 1. Introduction

In the context of deepening economic globalization, water transportation has emerged as a pivotal mode of conveyance, facilitating nearly 90% of the global bulk trade freight volume and significantly propelling the development of both world and regional economies [1]. China’s shipping industry has experienced notable growth, with a steady increase in the number of ships, particularly in inland rivers. While ship transportation has played a crucial role in reducing social logistics costs, it has concurrently elevated the risk of ship collisions, including those with bridges, and other navigation accidents, thereby posing a substantial threat to personal and property safety [2]. Consequently, enhancing the safety of ship navigation in dense traffic, intricate ports, or waterways has become a paramount concern.

In recent years, the global trend towards intelligent shipping has gained momentum, prompting countries to actively explore intelligent water transportation. Various intelligent perception technologies, such as binocular vision and communication equipment, are employed to collect data on ships, navigation environments, ports, and docks [3]. Advanced technologies like big data, machine learning, image processing, and pattern recognition are harnessed to process, analyze, evaluate, and make decisions based on this information, ultimately aiming to improve the safety of ship navigation [4, 5].

Currently, a series of deep learning-based object detection methods have been widely applied in various fields of maritime vessels. Jiang [6] proposed a new learning framework called Multi-Graph Learning Neural Network (MGLNN), used for multi-graph learning and multi-view semi-supervised classification. Roy [7] introduced the WilDect-YOLO model for real-time detection of endangered wildlife, achieving an mAP of 96.89% at a detection rate of 59.20 FPS and an F1 score of 97.87%, surpassing the current state-of-the-art models. Arunabha [8] developed an efficient object localization model, DenseSPH-YOLOv5s, for precise classification and localization of eight different types of road damages. Roy [9] proposed a high-performance real-time fine-grained object detection framework, which efficiently detects four different diseases in tomato plants in various challenging environments. Talib [10] introduced the YOLOv8-CAB model for weak feature handling, context information preservation, and effective feature fusion, achieving an average detection accuracy of 97%, a 1% improvement over traditional models. In the maritime domain, Lu [11] proposed a ship recognition method that combines compressive sensing and saliency detection. Liang [12] introduced an improved Faster R-CNN to enhance the accuracy and efficiency of ship detection. Yu [13] utilized DIOU based on the R-CNN object detection algorithm to replace IOU and weighted it through confidence scoring, enhancing the model’s detection capability under ship density conditions. Jiang [14] presented a lightweight real-time ship detection model called YOLOSeaShip, this model achieves ship detection by segmenting the image into grids and predicting the presence of ships in each grid, Compared to other ship detection models, YOLOSeaShip has faster detection speed and higher accuracy. The above research achieved good results in terms of detection accuracy, but the model has a large number of parameters and high complexity. In order to effectively execute ship recognition tasks, it is necessary to provide a lightweight model with minimal hardware performance requirements. In recent years, maintaining a balance between the accuracy and detection speed of network models has received widespread attention from many scholars, leading to the proposal of a series of lightweight and efficient network architectures such as MobileNet [15–17], GhostNet [18], and EfficientNet [19]. Zheng [20] used the MobileNetV1 lightweight network to replace the original feature extraction backbone network in YOLOv4, presenting a lightweight ship detection model named MobileNetV1-YOLOv4, which enhances the detection speed in ship ranging and recognition stages. Zou [21] combined MobileNetV2 with the SSD algorithm, utilizing the MobileNetV2 lightweight network to extract features, thereby saving training time and computational resources.

Stereo vision technology constitutes a pivotal branch in the field of computer vision, enabling the recognition, localization, and depth estimation of objects. Solak [22] employed two cameras positioned along the same axis to accurately estimate distances in real-time, measured in pixels and centimeters, between a robot and an object or between two different objects. In their experimental study, they conducted 150 measurements for distances ranging from 60cm to 140cm, achieving an accuracy of over 90% in their measurements. Zhang [23] established a target distance measurement model based on deep learning and stereo vision, calibrating camera intrinsic and extrinsic parameters, using the Faster R-CNN algorithm for object recognition, and applying stereo vision to obtain depth information of target objects. However, this study focused solely on detecting single obstacles. Zheng [24] explored a novel ship positioning system using stereo vision technology, employing the SURF algorithm for feature point matching to achieve pixel-level accurate matching in camera video images, thereby enhancing system accuracy. But their approach exhibited a complex structure and high computational requirements. Solak [25] proposed a new distance estimation method for indoor mobile robot platforms based on hybrid stereo vision, calculating both Manhattan and Euclidean distances for objects not on the same line as the robot, achieving an accuracy of over 98%.

In various fields such as digital signal processing, navigation, guidance, and control, the Kalman filtering algorithm has become a key technology due to its excellent performance and wide applicability. Hao [26] presented an improved Kalman filtering localization method for non-line-of-sight (NLOS) environments, eliminating clock synchronization issues and improving localization accuracy and reliability. Ali [27] applied Kalman filtering to track target vehicles, combining it with the YOLOv3 algorithm for target detection. They continuously updated target vehicle position and velocity information using residuals between measured and predicted values, achieving more accurate and stable tracking. Chakraborty [28] utilized the Kalman filtering algorithm for boundary tracking in lane detection, enhancing lane line position accuracy and stability by handling image noise and uncertainty caused by low light, heavy rain, etc. Therefore, integrating these techniques has great potential to enhance depth perception capabilities for river ship navigation.

In summary, in the field of ship detection, the main issue that urgently needs to be addressed is the poor real-time performance of existing methods when faced with large-scale parameters and high computational complexity. Additionally, in ship ranging, the instability and significant errors in measurement data caused by environmental factors pose challenges. Traditional ranging algorithms struggle to provide stable distance estimation results, posing a potential threat to navigation safety. To address these issues, this paper proposes a lightweight ship detection method and a high-precision, high-robustness binocular ranging approach. The aim is to enhance the environmental perception capabilities and navigation safety of inland vessels.

The remaining sections of this paper are organized as follows: Section 2 provides a brief introduction to the MS-YOLOv5s algorithm proposed in this paper. Section 3 elaborates on the principles of stereo vision. Section 4 combines stereo vision with the Kalman filtering algorithm and proposes the Binocular Kalman Filter Fusion Ranging Algorithm. Section 5 conducts ship target recognition ranging experiments to validate the effectiveness of this approach.

The main contributions of this paper are as follows:

1. A lightweight network-based ship target detection algorithm, MS-YOLOv5s, was proposed, achieving a balance of high speed and high accuracy.
2. A binocular ranging algorithm combined with Kalman filtering was introduced, enhancing the stability and accuracy of measurement results.
3. The proposed ship target recognition and depth estimation technology based on binocular vision provides technical support and solutions for improving the navigation safety and environmental perception capabilities of inland waterway vessels.

## 2. Lightweight ship target detection model

### 2.1 MobileNetV3 model

**Fig 1:**
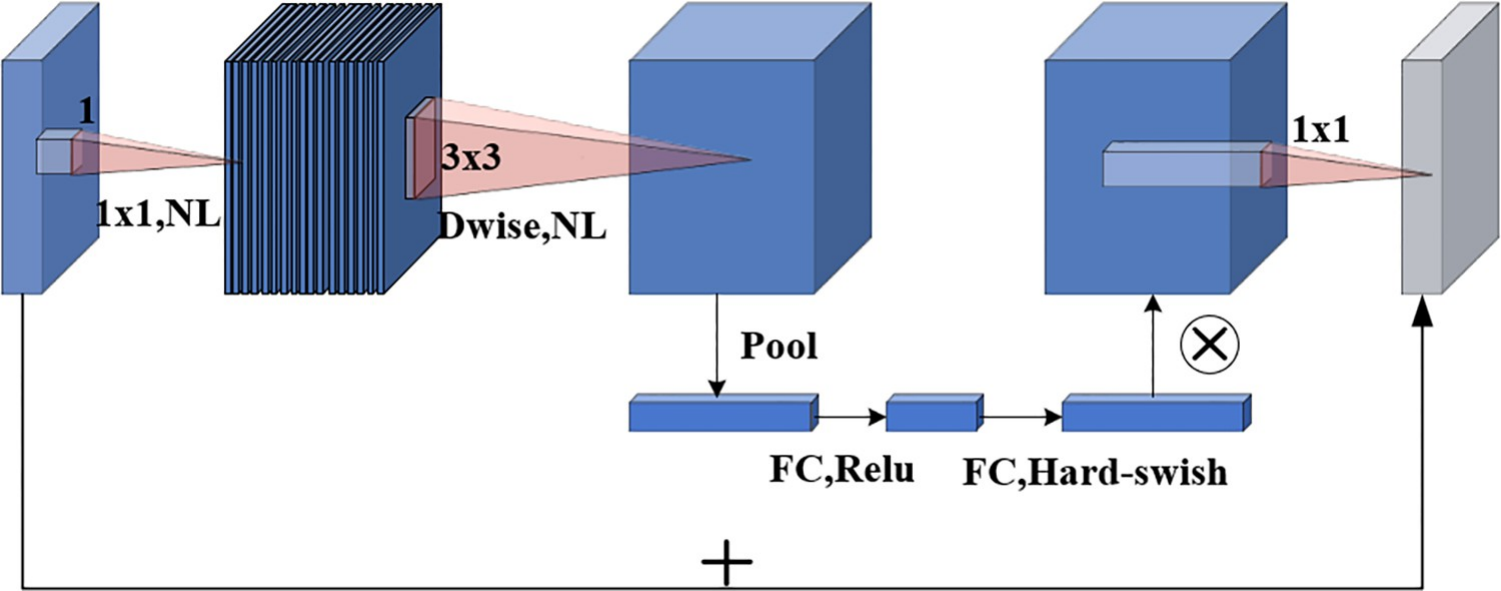
Bottleneck structure diagram.

MobileNetV3 [29] represents the latest advancement within the MobileNet series, renowned for its compact size, reduced trainable parameters, and decreased computational demands, rendering it particularly well-suited for integration into mobile devices. This iteration of the network comprises two variants: large and small, with the latter exhibiting diminished model complexity compared to its larger counterpart. The principal distinction between the two versions lies in the composition of Bottleneck modules and their respective internal parameters, notably the number of channels. Specifically, the large version encompasses 15 Bottleneck modules, while the small version comprises 11. Central to the architecture is the Bottleneck module, depicted in Fig 1, which incorporates depthwise separable convolution, SE channel attention mechanism, and residual connections, constituting fundamental elements of the network’s design.

**Fig 2:**
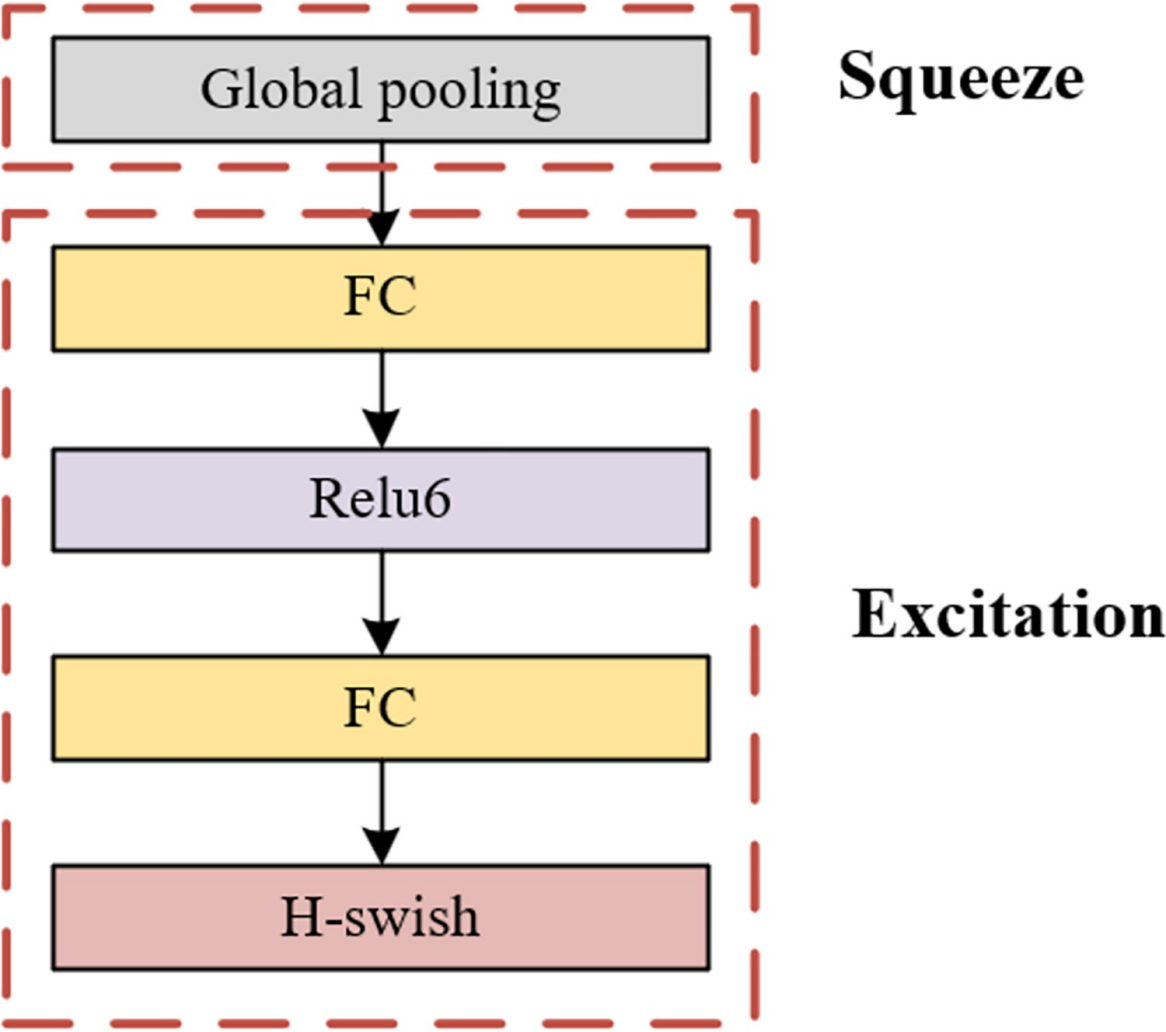
SE module structure diagram.

MobileNetV3 represents a notable advancement by incorporating the SE module between depthwise convolution and pointwise convolution, as elucidated in Fig 2. The SE attention mechanism serves as a pivotal tool for augmenting the network’s receptive field, comprising two fundamental steps: Squeeze and Excitation. During the Squeeze step, each channel of the feature map is compressed into a scalar representation. Subsequently, in the Excitation step, these scalars are weighted utilizing a fully connected layer, thereby determining the significance of various channels. This amalgamation of depthwise convolution and attention mechanism introduces a novel approach to designing lightweight neural networks, facilitating the attainment of high-precision image recognition and processing within resource-constrained environments.

### 2.2 MS-YOLOv5s model structure

**Fig 3:**
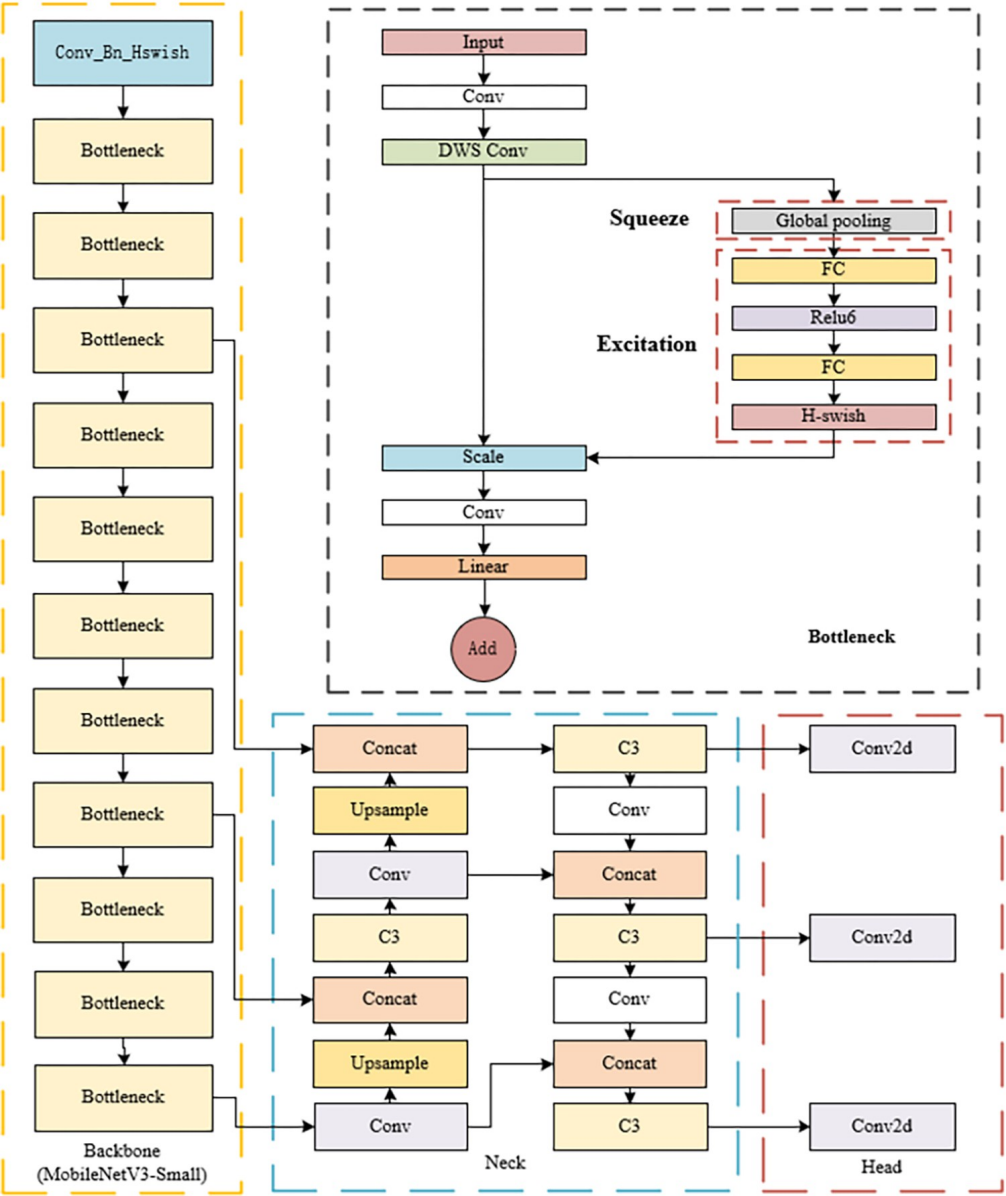
MS-YOLOv5s network structure.

While the YOLOv5s algorithm showcases commendable performance in object detection endeavors, its practical application in ship target detection tasks necessitates further enhancement. As depicted in Fig 3, this study endeavors to tackle the challenges stemming from the high parameter counts and complexity inherent in the YOLOv5s model, thereby mitigating its suboptimal real-time performance and elevated device resource requirements. To this end, a lightweight and efficient feature extraction network is devised by integrating the Small version of MobileNetV3 into the backbone network of MS-YOLOv5s. This substitution replaces the CSPDarknet53 feature extraction backbone network employed in YOLOv5s, thereby optimizing the model for ship target detection applications.

The MobileNetV3-Small model consists of a total of 16 layers, with the last 4 layers representing the main network structure for classification. Therefore, only the first 12 layers of MobileNetV3-Small, which possess feature extraction capabilities, are retained to replace the original backbone of the YOLOv5s model, achieving model lightweighting and improving the computational speed of the algorithm in mobile and embedded device environments. The structure and parameter settings of the backbone network are illustrated in Table 1.

**Table 1:** The detailed structure of the backbone.

Operator	Stride	Output	Expansion factor	SE	NL
Conv_Bn_Hswish	2	16	—	X	H-swish
Bottleneck*3	[2,2,1]	[16,24,24]	[16,72,88]	**√**	ReLU
Bottleneck*5	[2,1,1,1,1]	[48, 48, 48, 48, 48]	[96,248,248,128,144]	**√**	H-swish
Bottleneck*3	[2,1,1]	[96,96,96]	[288,576,576]	**√**	H-swish

## 3. Principle of binocular stereo vision

**Fig 4:**
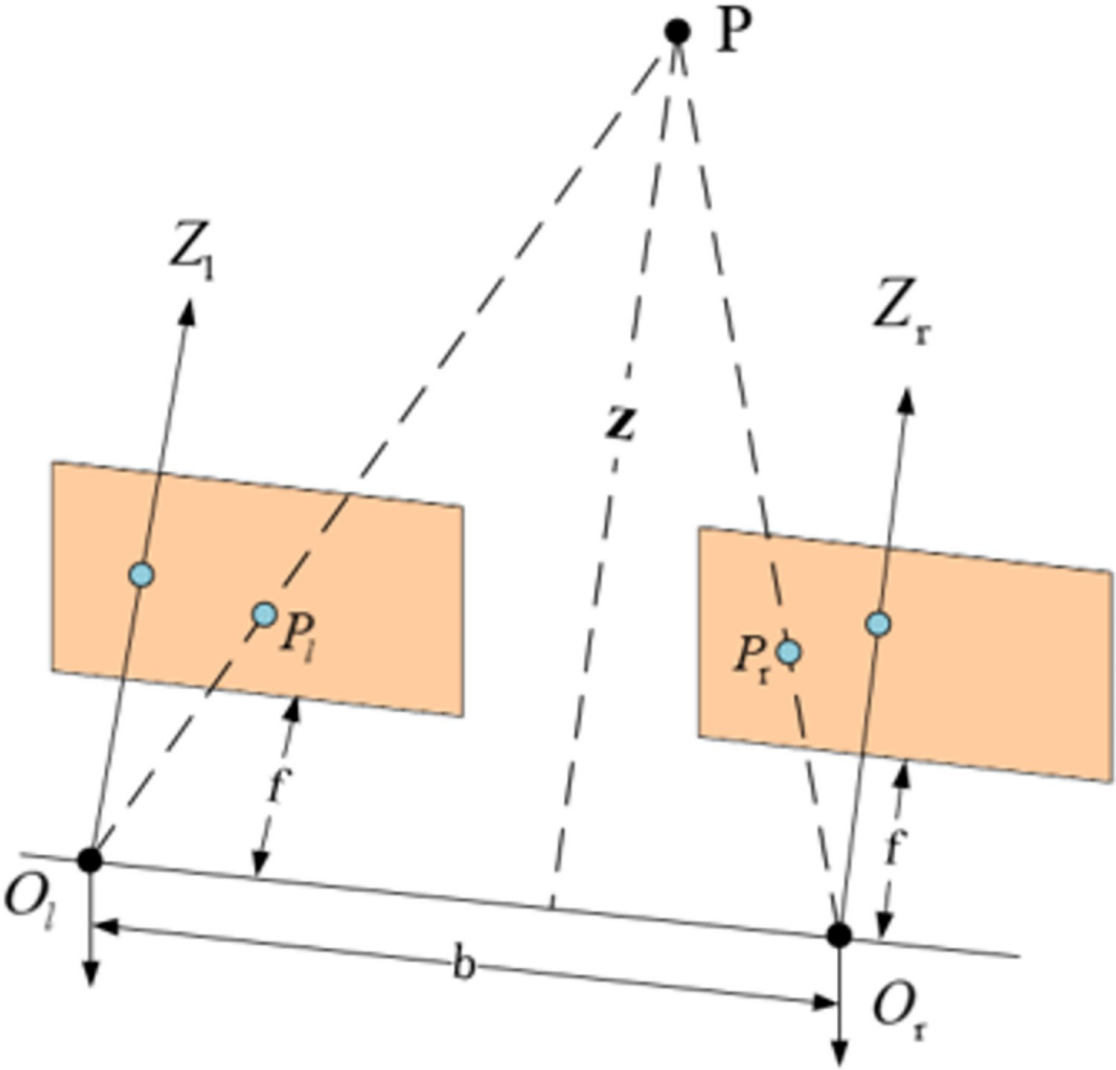
System model for binocular stereo vision ranging.

Binocular stereo vision ranging primarily involves simultaneously capturing images of the same object using two different cameras. Subsequently, three-dimensional information is extracted from these two images to facilitate three-dimensional reconstruction [20]. The parallax value, a measure of the displacement of corresponding points between the left and right images, is then converted into distance information through the triangulation method. This conversion allows for accurate distance measurement. The system model depicting this principle is illustrated in Fig 4.

In the depicted figure, *P* represents the target under measurement. *O*_*l*_ and *O*_*r*_ denote the optical centers of the left and right cameras, respectively. The distance between the optical centers of the left and right cameras, referred to as the baseline distance, is denoted as *b*. Additionally, *f* represents the focal length of the camera. *P*_*l*_ and *P*_*r*_ represent the coordinates of point *P* in the image coordinate system of the left and right cameras. *Z* signifies the vertical distance from point *P* to the cameras.

**Fig 5:**
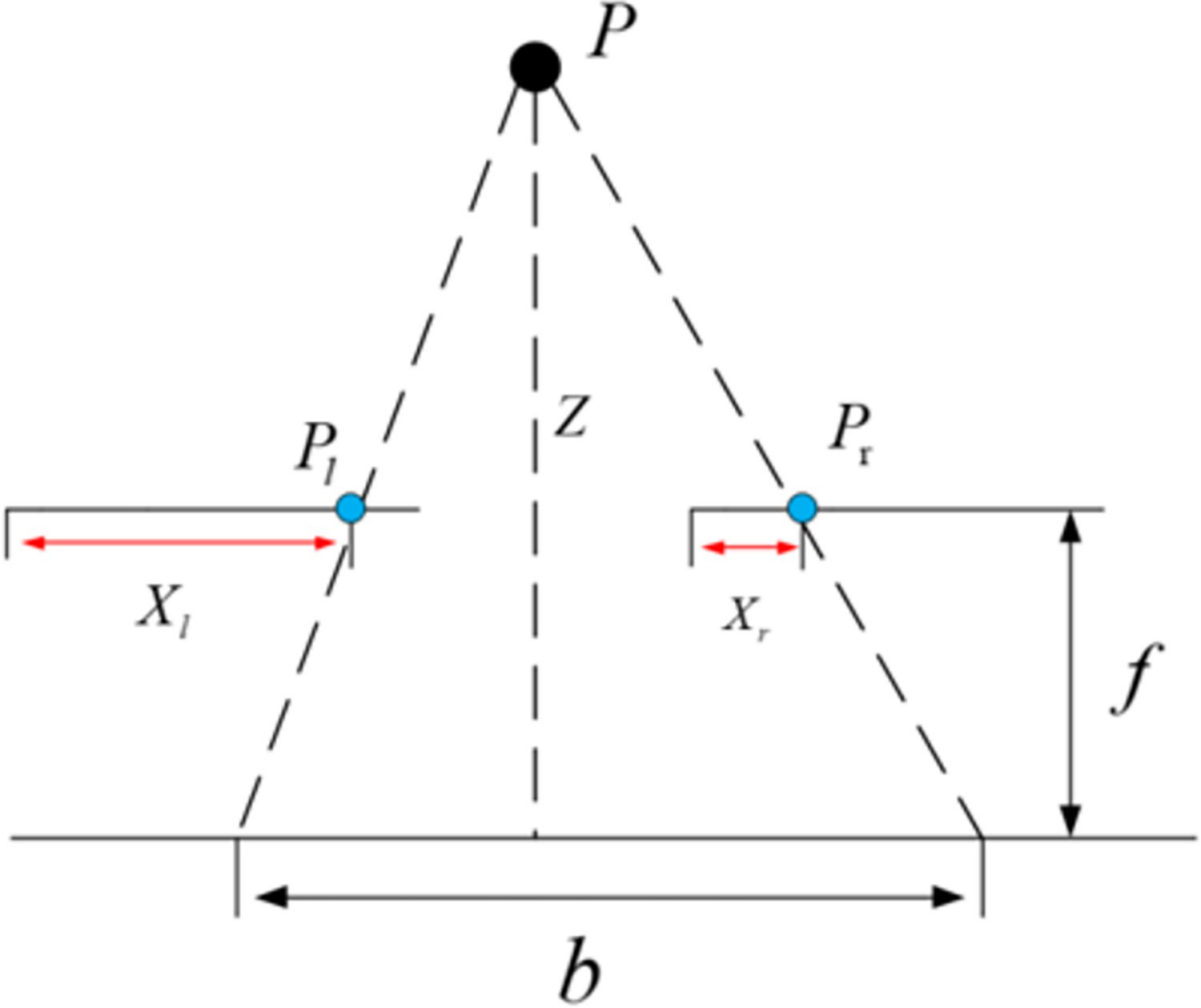
Two-dimensional projection of binocular stereo vision system model.

When projecting the aforementioned diorama onto a two-dimensional plane, the result is illustrated in Fig 5.

Based on the principle of similar triangles, we can derive Eq (1):

$$\frac{b-(X_{l}-X_{r})}{b}=\frac{Z-f}{Z}$$ (1)

From this, the expression for the distance *Z* between the target under measurement and the camera can be derived as follows:

$$Z=\frac{fb}{X_{l}-X_{r}}=\frac{fb}{d}$$ (2)

In Eq (2), *d* represents the parallax between the left and right cameras, calculated as *d* = *X*_*l*_−*X*_*r*_. The focal length *f* and the baseline distance *b* can be determined through camera calibration. Consequently, distance information can be accurately obtained by measuring the parallax *d*.

## 4. Binocular Kalman filter fusion ranging model

### 4.1 Binocular calibration

**Fig 6:**
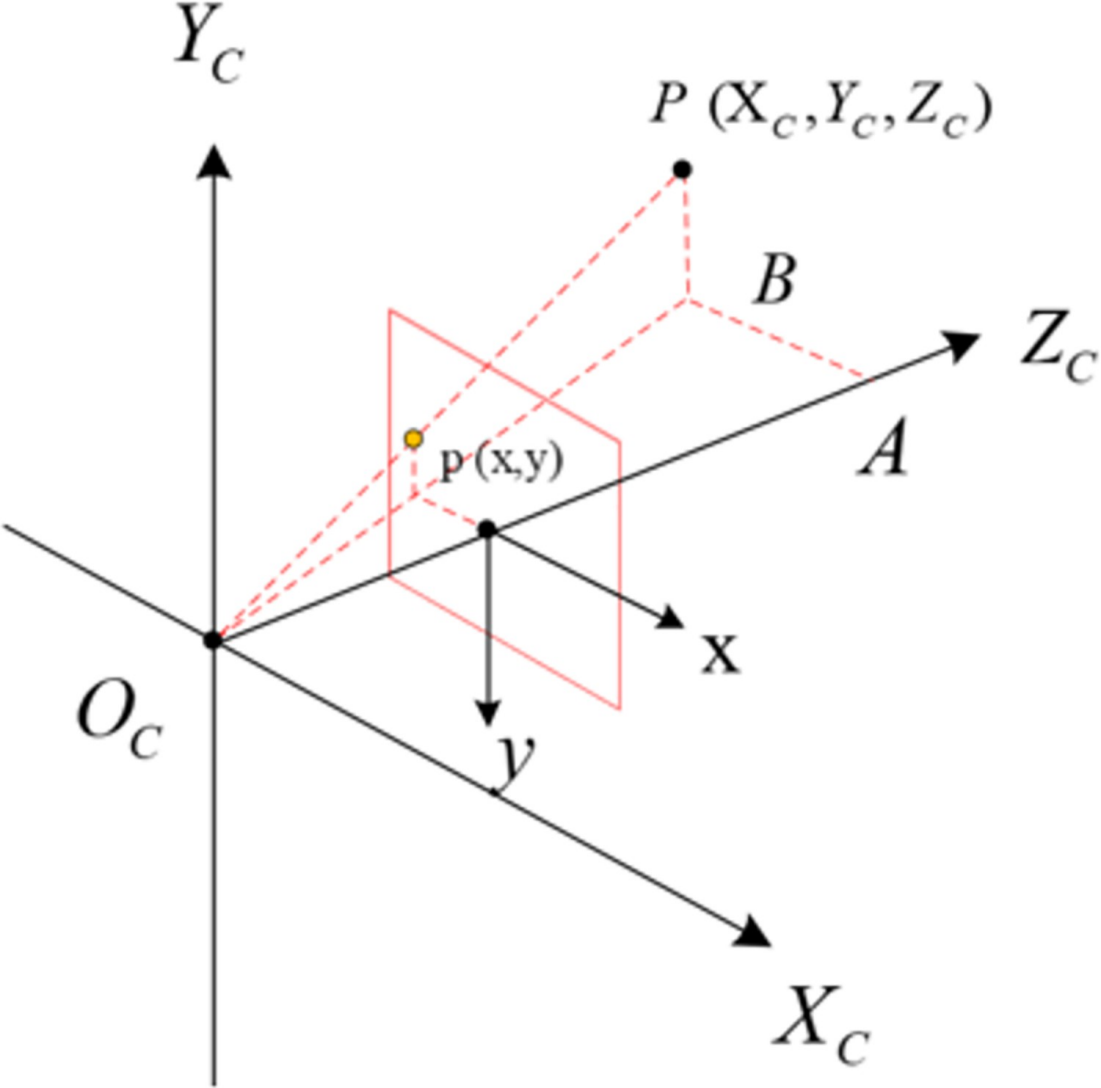
Conversion between camera coordinate system and image coordinate system.

In camera imaging, the ideal pinhole imaging model is commonly employed. During image capture, light reflected from the real object is focused and projected onto the imaging screen via the camera lens. This intricate process entails the mapping of points in three-dimensional space to a two-dimensional image through a sequence of coordinate transformations, as depicted in Fig 6.

**Fig 7:**
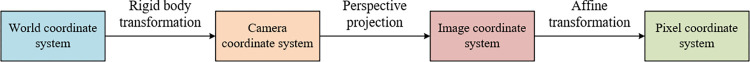
Relation diagram of coordinate system transformation.

This system mainly involves four coordinate systems: World Coordinate System, Camera Coordinate System, Image Coordinate System, and Pixel Coordinate System. According to the principle of pinhole imaging, in Fig 7, assuming a point *P*_*w*_(*X*_*w*_, *Y*_*w*_, *Z*_*w*_) in the three-dimensional world coordinate system, its projection coordinates in the camera coordinate system are *P*_*c*_(*X*_*c*_, *Y*_*c*_, *Z*_*c*_). The corresponding coordinates in the image coordinate system are *P*(*x*, *y*), and in the pixel coordinate system, they are *P*(*u*, *v*). *O*_*c*_ represents the position of the optical center of the cameras, and *O*_*c*_*Z*_*c*_ denotes the optical axis of the cameras.

The relationships among these four coordinate systems are depicted in Fig 7. We can transform a point from the world coordinate system to the pixel coordinate system using Eq (3), which is expressed as follows:

$$\left(\begin{array}{c} u\\ v\\ 1 \end{array})=\frac{1}{Z_{c}}(\begin{array}{cccc} \frac{f}{dx} & 0 & u_{0} & 0\\ 0 & \frac{f}{dy} & v_{0} & 0\\ 0 & 0 & 1 & 0 \end{array})(\begin{array}{cc} R & T\\ 0^{T} & 1 \end{array})(\begin{array}{c} X_{w}\\ Y_{w}\\ Z_{w}\\ 1 \end{array}\right)$$ (3)

$$Z_{c}(\begin{array}{c} u\\ v\\ 1 \end{array})=M_{1}M_{2}(\begin{array}{c} X_{w}\\ Y_{w}\\ Z_{w}\\ 1 \end{array})$$ (4)

In Eq (4), *dx* and *dy* represent the physical measurement size of a unit pixel in the x and y axes, respectively. (*u*_*0*_, *v*_*0*_) denotes the center position of the image, *M*_*1*_ represents the camera internal parameter matrix, and *M*_*2*_ represents the camera external parameter matrix.

### 4.2 Binocular stereo matching

Stereo matching technology plays a pivotal role in establishing the corresponding relationship between points in the left and right images, thereby deriving parallax. Subsequently, depth and three-dimensional information of the object under scrutiny are acquired based on the projection model. The Semi-Global Block Matching (SGBM) algorithm [30, 31] enhances the Block Matching (BM) algorithm’s single-direction matching accuracy by incorporating multi-path constraint cost aggregation. Moreover, it introduces penalty coefficients to accommodate inclined and curved surfaces while preserving discontinuity.

Post-processing within the SGBM algorithm encompasses uniqueness detection and left-right consistency detection, aimed at smoothing parallax and rectifying errors stemming from left-right occlusion. The initial parallax map is obtained by employing the SGBM algorithm for individual pixels. Subsequently, the optimal parallax value for each pixel is determined by resolving the minimum value of the global energy function, as delineated in Eq (5) [30]:

$$E(D)=\sum_{p}\left(C(p,D_{p}\right)+\sum_{q\in N_{p}}p_{1}I[|D_{p}-D_{q}|=1]+\sum_{q\in N_{p}}p_{2}I[|D_{p}-D_{q}|>1])$$ (5)

**Fig 8:**
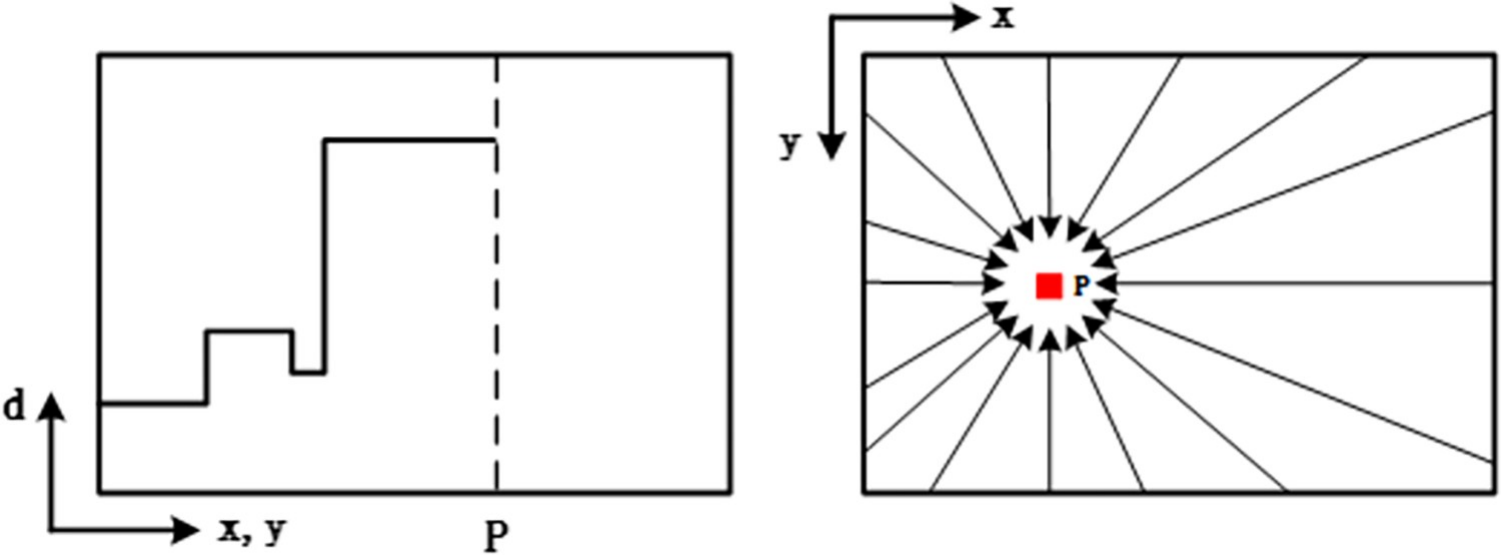
Energy aggregation in Semi-Global Block Matching (SGBM) algorithm.

In Eq (5), the global energy function corresponding to the disparity map *D* is denoted by *E*(*D*). Pixel points are represented by *p* and *q*, where *N*_*p*_ denotes the 16 neighboring pixels of pixel point *p*. The cost of pixel point *p* at disparity value *D*_*q*_ is denoted by *C*(*p*,*D*_*p*_). Penalty coefficients for the difference between the disparity values of adjacent pixels and the pixel point p are denoted as *P*_*1*_ and *P*_*2*_ for differences equal to and greater than 1, respectively. The SGBM algorithm aims to establish a global Markov energy equation by constraining one-dimensional paths in multiple directions on the image. The final matching cost for each pixel is the summation of information along all paths. Disparity selection for each pixel is determined by Winner Takes All (WTA), and the aggregation of energy in multiple directions is illustrated in Fig 8.

### 4.3 Binocular Kalman filter fusion ranging algorithm

Due to the susceptibility of distance calculations in complex environments to noise interference, Kalman filters [26] can effectively remove sudden variations in signals during the estimation process based on previous signals. In the proposed binocular ranging framework, Kalman filtering is employed to fuse the measurement results of the binocular ranging system, making the estimates more stable and closer to the true values. The state-space model of the Kalman filter algorithm is represented by Eqs (6 and 7):

X(k+1)=ϕX(k)+ΓW(k) (6)

Y(k)=HX(k)+V(k) (7)

Here, *k* is discrete time, ***X***(*k*) represents the system state at time *k*, and ***Y***(*k*) is the observed signal for the corresponding state. *W*(*k*) is the input white noise, and ***V***(*k*) is the observation noise. *ϕ* is the state transition matrix, ***Γ*** is the noise drive matrix, and ***H*** is the observation matrix. In Eq (8), ***Z***_*p*_ serves as the input to the Kalman filter:

$$\hat{X}(k|k)=Z_{p}$$ (8)

Eqs (9–11) represents the one-step prediction formula for the state:

$$\hat{X}(k+1|k)=\phi \hat{X}(k|k)$$ (9)

$$\hat{X}(k+1|k+1)=\hat{X}(k+1|k)+K(k+1)\varepsilon (k+1)$$ (10)

$$\varepsilon (k+1)=Y(k+1)-H\hat{X}(k+1|k)$$ (11)

Eq (12) represent the filter gain matrix:

$$P(k+1|k)H^{T}{\left[HP(k+1|k)H^{T}+R\right]}^{-1}$$ (12)

Eq (13) represent the one-step prediction covariance matrix:

$$P(k+1|k)=\phi P(k|k)\phi^{T}+{\mathrm{\Gamma Q\Gamma}}^{T}$$ (13)

The covariance matrix update is represented by Eq (14):

$$P(k+1|k+1)=[I_{n}-K(k+1)H]P(k+1|k)$$ (14)

In the expressions, ***P***(*k*+1|*k*+1) and ***P***(*k*+1) represent the covariance matrices corresponding to the estimation results, respectively. *R* is the measurement noise matrix, and *K* is the Kalman filter gain, determined by the covariance matrix and the measurement error matrix. The larger the *K* value, the more trust is placed in the observed value, while the smaller the *K* value, the more trust is placed in the predicted value. ***X***(*k*) is the optimal estimation result of the previous state, and ***Z***_*p*_ is the data optimization result of the current time binocular ranging algorithm.

The process of binocular Kalman filter fusion ranging starts with the acquisition of images from the stereo high-definition cameras, capturing both left and right images. Firstly, the MS-YOLOv5 model is utilized to detect ship targets in the left image, identifying and locating the positions of the target ships.

**Fig 9:**
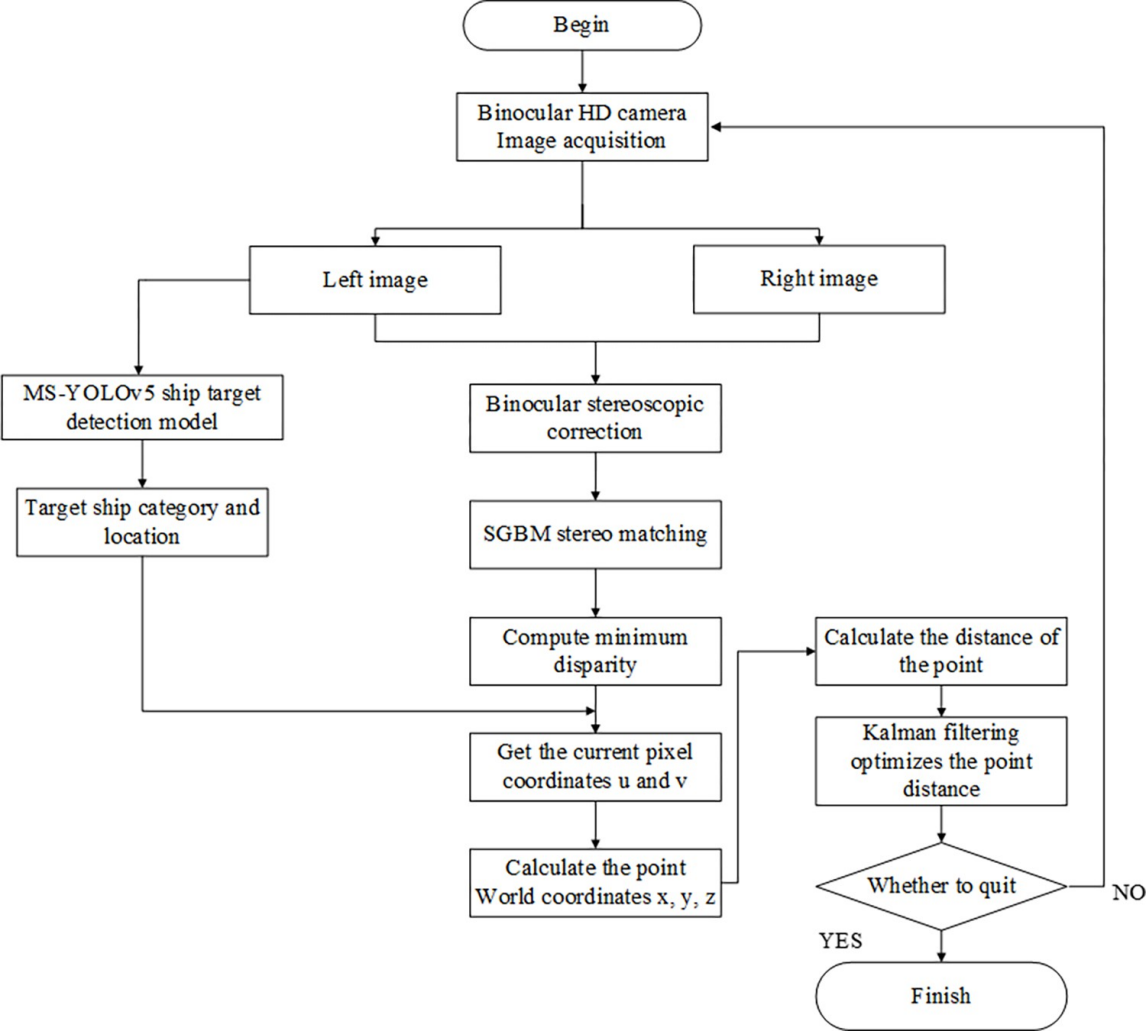
Flow chart of binocular Kalman filter fusion ranging.

Subsequently, stereo rectification is performed on the left and right images to ensure image alignment and reduce errors. Next, SGBM algorithm is employed for stereo matching, calculating the minimum disparity value of the target ships. Based on this, the pixel coordinates of the current detected targets in the images are obtained. Using the geometric relationships of stereo vision, the positions of the targets in the world coordinate system are calculated, determining the distances between the target ships and the cameras. Then, the Kalman filtering algorithm is applied to optimize the calculated distances. By iteratively optimizing the measurement data, the algorithm gradually approaches the true values, enhancing the accuracy and stability of distance measurement. Kalman filtering technology models the uncertainty of binocular vision measurements, combining it with the system’s dynamic model for state prediction. This effectively eliminates measurement errors and instability, making the ranging results more reliable and accurate. The specific ranging process of this paper is illustrated in Fig 9.

## 5. Experiment and analysis

### 5.1 Experimental environment and evaluation metrics

The experiment was conducted on the Windows 11 operating system, utilizing a binocular stereo camera model HBVCAM-4M2142HD V22. The hardware setup comprised an AMD R5-5600 CPU @ 4.40GHz processor and an NVIDIA 4060Ti GPU. PyCharm served as the development environment, with the experimental software stack incorporating the TensorFlow deep learning framework along with the OpenCV library.

To comprehensively assess the quality of ship target recognition and detection outcomes, this study employs evaluation metrics including precision (P), recall rate (R), mean average precision (mAP), frames per second (FPS), and other relevant indicators, as delineated in Eqs (15–18).


$$P=\frac{TP}{TP+FP}$$ (15)

$$R=\frac{TP}{TP+FN}$$ (16)

$$mAP=\frac{\sum_{i=1}^{k}AP_{i}}{k}$$ (17)

$$FPS=\frac{n}{T}$$ (18)

*TP* represents true positive samples predicted by the model as positive, *TN* represents true negative samples predicted by the model as negative, *FP* represents false positive samples predicted by the model as positive, *FN* represents false negative samples predicted by the model as negative, and *k* denotes the number of classes in the current recognition task.

### 5.2 Calibration and calibration experiments

Camera calibration constitutes a fundamental step in binocular vision ranging technology, and the precision of the calibration outcome profoundly impacts ranging effectiveness. Several factors, including camera structural parameters and environmental conditions, can influence calibration results. For this experiment, the baseline distance between cameras was set at 120mm, with the camera maintained parallel to the ground.

To ensure experimental accuracy, images were collected at distances of 300mm, 400mm, 500mm, 600mm, and 700mm from the camera calibration board. The Zhang Zhengyou calibration method [26] was employed, involving the capture of images at varying poses and angles. A black and white checkerboard calibration board, measuring 8×7 grids with each grid spanning 25mm, was utilized.

A total of 38 sets of calibration plate images were captured by the binocular camera from diverse angles, with 32 sets of images selected following screening. The calibration results are presented in Table 2 below.

**Table 2:** Calibration parameters of binocular camera.

Parameter	Left Camera	Right Camera
Internal parameter matrix	$\left[\begin{array}{ccc} 514.7514 & 0 & 307.4342\\ 0 & 511.0474 & 247.9709\\ 0 & 0 & 1 \end{array}\right]$	$\left[\begin{array}{ccc} 512.1546 & 0 & 313.3743\\ 0 & 509.2002 & 250.6284\\ 0 & 0 & 1 \end{array}\right]$
Distortion coefficient matrix	[‐0.03230.11740.0018‐0.00840]	[‐0.06080.28630.0025‐0.00750]
Rotation matrix	$\left[\begin{array}{ccc} 1 & 0 & 0.0015\\ 0 & 1 & 0.0033\\ 0.0015 & 0.0033 & 1 \end{array}\right]$	
Translation vector	[−120.64−0.01951.3086]	

**Fig 10:**
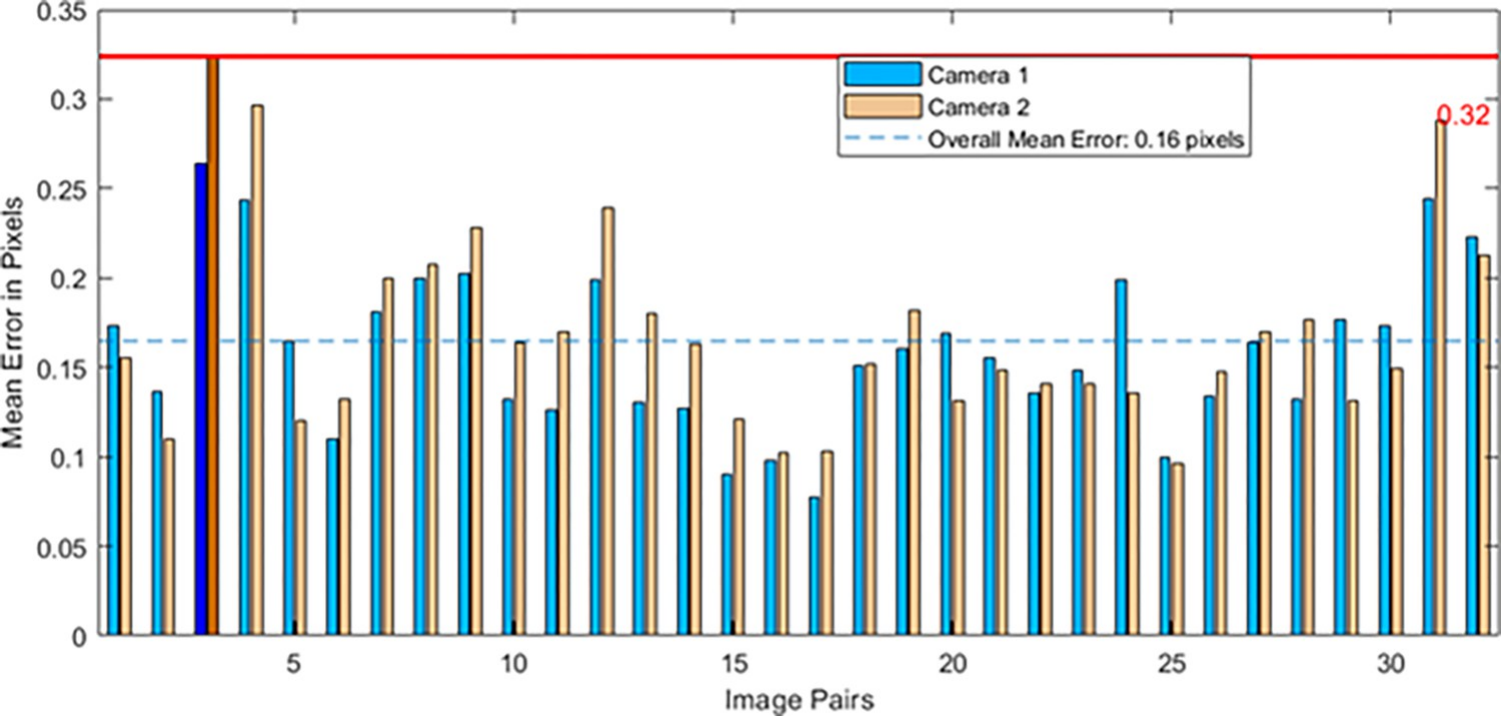
Error analysis of camera reprojection.

In the Table 2, it is evident from the internal parameter matrix that the focal lengths of the cameras are nearly identical. Moreover, the rotation matrix bears a resemblance to the identity matrix, suggesting that the two cameras are fundamentally parallel. At the same time, we obtained an analysis of camera reprojection error, as shown in Fig 10.

The figure above illustrates the error analysis of camera reprojection. The maximum calibration error is 0.32 pixels, and the average error is 0.16 pixels, both of which are less than 1 pixel, meeting the experimental standard. The obtained parameters are suitable for stereoscopic correction of images.

### 5.3 Ship target recognition experiment

During the quantitative evaluation phase of model performance, we employed the publicly available SeaShips (7000) dataset [32] to assess the effectiveness of the enhanced model, this dataset contains 7000 images of 6 common types of ships. Following 100 iterations, the model’s loss function continuously decreased and reached convergence, resulting in a well-trained model.

**Fig 11:**
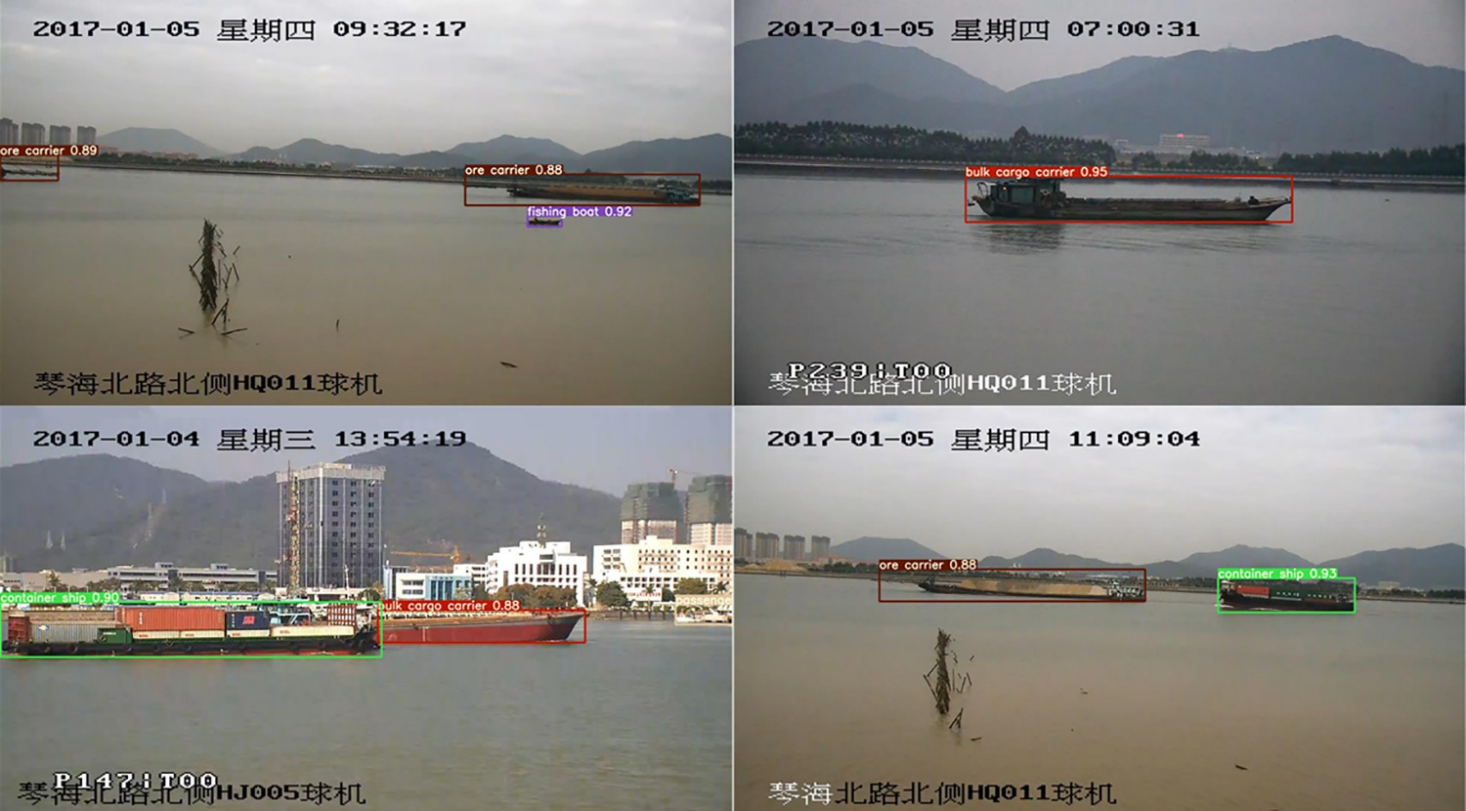
Ship detection performance of the MS-YOLOv5s model.

MS-YOLOv5s visualization results on different types of ships in the test set are shown in Fig 11. The model can accurately identify targets of various ship types with high precision.

In this section, we evaluate and analyze the proposed MS-YOLOv5s model in comparison with YOLOv5s, YOLOv7, and YOLOv8 under the same experimental conditions. The comparison focuses on parameters, FLOPs, and various evaluation metrics on the validation set. The results are presented in Table 3.

**Table 3:** Comparison of different models.

Model	Precision	Recall	mAP	FPS	Parameters	FLOPs
YOLOv5s	0.971	0.981	0.986	24.33	7.03MB	15.8G
YOLOv7	**0.983**	**0.982**	**0.992**	31.05	37.22MB	105.2G
YOLOv8	0.975	0.961	0.987	30.99	4.01MB	8.2G
**MS-YOLOv5s** **(ours)**	0.968	0.977	0.984	**50.28**	**3.55**MB	**6.3G**

This study employs YOLOv5s as the base model. As illustrated in Table 3, while its precision is slightly lower than the other three models, YOLOv7’s parameter size is 37.22MB, more than ten times that of MS-YOLOv5s. The FLOPs of YOLOv8 exceed those of MS-YOLOv5s by 1.9G, and the parameters of YOLOv5s are 6.99MB larger, over twice the size of MS-YOLOv5s.

Compared to YOLOv5s, YOLOv7, and YOLOv8, MS-YOLOv5s achieves a reduction in FLOPs by 60.1%, 94%, and 23.2%, respectively, and an increase in FPS by 106.7%, 61.9%, and 62.2%, respectively. This demonstrates that our proposed model boasts higher FPS and smaller parameter sizes, rendering the model more lightweight. The results indicate that the proposed MS-YOLOv5s model significantly reduces network parameters and enhances computational speed. With high recognition accuracy guaranteed, it possesses high real-time performance, exhibits good detection effects for different types of vessels, and can meet the detection requirements for various types of vessels in practical scenarios.

### 5.4 Ship target ranging experiment

**Fig 12:**
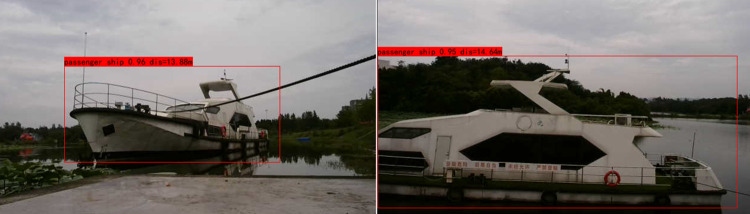
Target identification and ranging map.

In the experiment, the binocular Kalman filter fusion ranging algorithm was utilized to perform stereo matching on the left and right views of the binocular cameras for computing disparities. The minimum disparity value of all feature points of a single target was used to calculate the target’s depth. Taking a passenger ship as an example, this paper conducted target recognition ranging experiments by positioning the target ship at different distances from the binocular cameras. Partial ranging results are illustrated in Fig 12.

**Fig 13:**
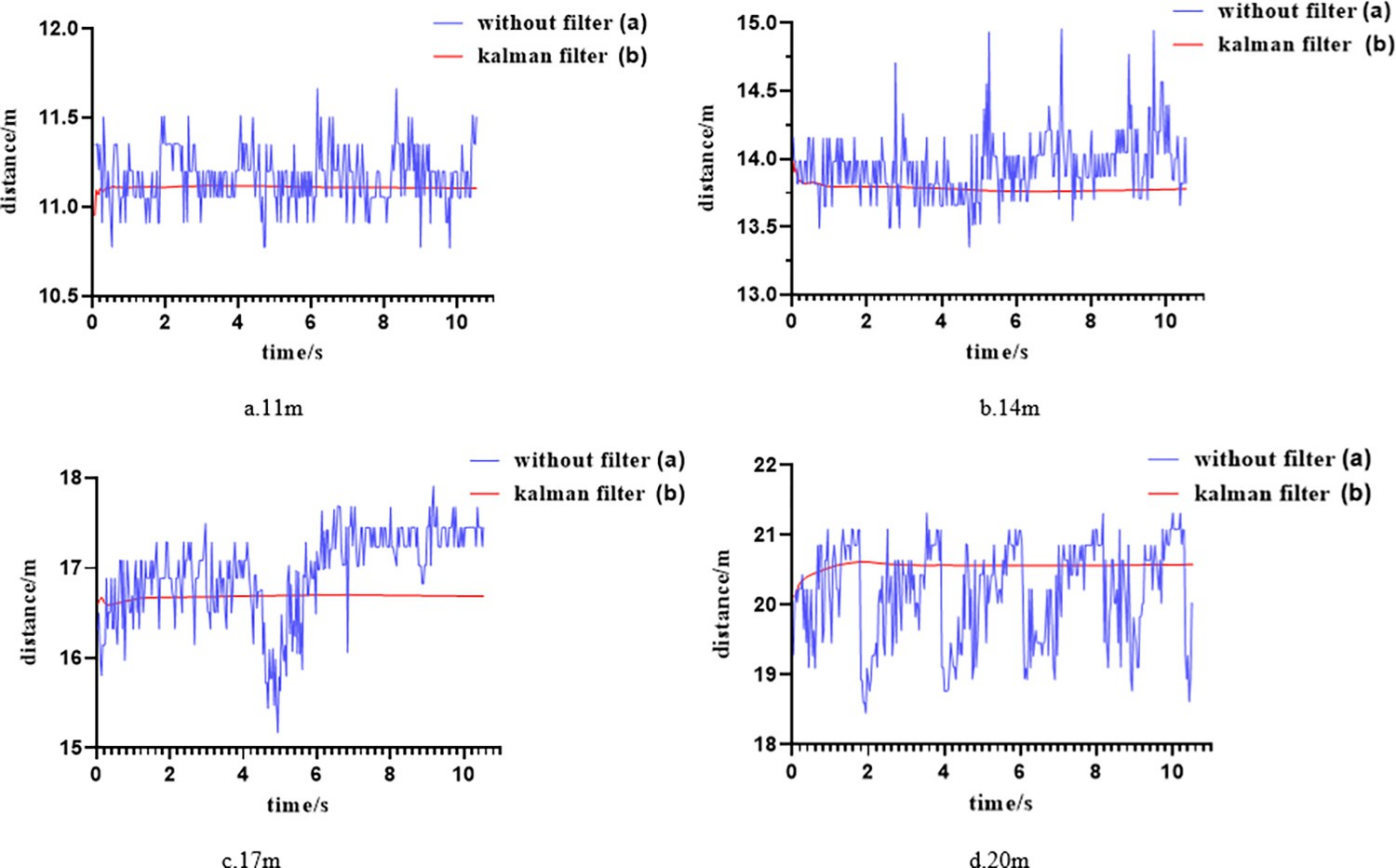
Distance estimation without filter and with Kalman filter.

Fig 13 depicts a comparison of distances between the target ship and the binocular camera at distances of 11m, 14m, 17m, and 20m using both the traditional ranging algorithm and the improved binocular Kalman filter fusion ranging algorithm. The traditional binocular ranging algorithm exhibits significant fluctuations in measured distances, as shown in Fig 13(A), Conditions such as illumination changes due to the sun or clouds, glare, reflection, obstruction, and other factors that may affect its performance. In contrast, the binocular Kalman filter fusion ranging algorithm proposed in this paper yields more stable distance data, as illustrated in Fig 13(B).

This study conducted multiple ranging experiments under the condition of stationary targets, analyzing the impact of integrating the Kalman filtering algorithm into the binocular ranging algorithm on the variance of ranging. By comparing the standard deviations of multiple sets of data before and after algorithm improvements, it was found that when the target ship was at different positions, the introduction of the Kalman filtering algorithm to process the measurement results resulted in a minimum standard deviation of 6.032 μm in ranging. This is a reduction of one order of magnitude compared to the traditional ranging algorithm, indicating a stronger robustness, as shown in Table 4.

**Table 4:** Comparison of standard deviations.

Experimental distances	Traditional binocular ranging algorithm	Binocular Kalman filter fusion ranging algorithm
11m	160.843um	6.032um
14m	234.321um	21.466um
17m	608.461um	21.391um
20m	707.182um	58.178um

To further validate the experiment’s effectiveness, a laser rangefinder was used for verification. The device has a range error of ±0.5m within 1000m. The comparative experimental analysis utilized the distances measured by the laser rangefinder as the standard, as shown in Table 5.

**Table 5:** Comparison of ranging results.

Laser ranging	Traditional binocular ranging	Binocular Kalman filter fusion ranging
1100cm	1122cm	1110cm
1400cm	1343cm	1376cm
1700cm	1779cm	1669cm
2000cm	1878cm	2059cm

The experimental results indicate that at target distances of 11m, 14m, 17m, and 20m, the proposed binocular Kalman filter fusion ranging algorithm significantly reduces the relative error compared to traditional ranging methods by 1.10%, 2.36%, 2.82%, and 3.15%, respectively. Specifically, the relative error is less than 1% at a target distance of 11m and less than 3% for distance of 20m. As the target distance increases, the difficulty in detecting and matching feature points also increases, affecting the disparity calculation results and increasing the measurement error. By optimizing the ranging results with the Kalman filter algorithm, environmental noise and system errors are effectively suppressed, resulting in enhanced robustness and improved ranging accuracy in complex and variable real-world application environments.

## 6. Conclusion

In response to the inadequacies of current environmental perception methods for inland waterway vessel navigation, this study improves binocular stereo vision and applies it to the identification and depth estimation of inland waterway vessels. The research is primarily divided into two stages: ship target detection and depth estimation. In the stage of ship recognition, addressing the issues of excessive parameters and computational load, poor real-time performance, and high memory requirements of existing ship detection models, we propose an improved algorithm, MS-YOLOv5s. By replacing the YOLOv5s model’s feature extraction network with the MobileNetV3 network, the parameter scale is significantly reduced. Experimental results show that while maintaining an mAP value of over 98%, the weight parameters of the MS-YOLOv5s backbone network are only 50% of those of YOLOv5s, markedly reducing computational demands. In the stage of ships’ depth estimation, to address the instability of distance measurements in complex environments, we propose a binocular Kalman filter fusion ranging algorithm. Experimental results indicate that the standard deviation of the distance measurement results processed by this algorithm is as low as 6.032μm, representing a reduction of an order of magnitude compared to traditional ranging algorithms, thus demonstrating greater robustness. Additionally, within a 20-meter range, the distance estimation error for target ships is controlled within 3%, indicating high depth estimation accuracy.

In future work, we plan to further improve the algorithm by combining different sensors or multiple feature descriptors to address this challenge. For example, integrating techniques that fuse visual features with laser radar data to enhance the accuracy of feature representation and matching for distant targets. This will effectively enhance the perceptual capabilities of vessels for navigation environments, thereby further enhancing the safety of inland waterway navigation.
